# Galectin-3 induces cell migration via a calcium-sensitive MAPK/ERK1/2 pathway

**DOI:** 10.18632/oncotarget.1786

**Published:** 2014-02-19

**Authors:** Xiaoge Gao, Vitaly Balan, Guihua Tai, Avraham Raz

**Affiliations:** ^1^ Department of Oncology, Karmanos Cancer Institute, Wayne State University, Detroit, MI, USA; ^2^ School of Life Sciences, Northeast Normal University, Changchun, PR China; ^3^ Present address: Everon Biosciences, Buffalo, NY

**Keywords:** galectin-3, calcium ions, protein kinase C, extracellular signal regulated protein kinase1/2, cell migration

## Abstract

The presence and level of circulating galectin-3 (Gal-3), a member of the galectin family, is associated with diverse diseases ranging from heart failure, immune disorders to cancer metastasis and serves as a biomarker of diagnosis and treatment response. However, the mechanisms by which exogenous Gal-3 affects pathobiology events remain elusive. In the current study, we found that exogenous Gal-3 slightly delays, while prolonging tyrosine phosphorylation of extracellular signal-regulated kinase 1/2 (ERK1/2) in HeLa cells through a calcium-sensitive and PKC-dependent signaling pathway. The activation was dependent on the sugar-binding properties of Gal-3, since the antagonist lactose could inhibit it. The sugar-binding motif of Gal-3 was required for the activation of ERK1/2. The activation of ERK1/2 was necessary for the initiation and induction of cell migration associated with the phosphorylation of paxillin. All the results presented in this study suggest a novel calcium-sensitive and PKC-dependent pathway through which circulating Gal-3 promotes cell migration and activating the ERK1/2. Taken together, the data depicted here propose a biological function and a target for the diseases' associated circulating Gal-3.

## INTRODUCTION

Gal-3 belongs to the animal galectin family, characterized by specific recognition and binding of β-galactosides (1). Gal-3 is the only chimeric protein in the galectin family based on its special structure. In addition to the evolutionarily conserved carbohydrate-recognition domain (CRD), it also has the N-terminal domain composed of a short N-terminal leader and a collagen-like internal repeating domain rich in glycine, tyrosine, and proline (2, 3). Gal-3 can be found in cytoplasm and nucleus, and it can also be secreted through non-classical pathways (4, 5). Many ligands of Gal-3 have been identified, including the glycoproteins from extracellular and intracellular proteins as well as glycolipids (6). While some binding interactions rely on the carbohydrate-binding domain of Gal-3, many Gal-3 binding partners depend on N-terminal protein-protein interaction. In the intracellular compartment, there are also many ligands, which bind mainly with Gal-3 through protein-protein interaction (7). Gal-3 is involved in multiple biological activities dependent on its wide distribution and abundant ligands, such as cell growth, cell differention, cell apoptosis, pre-mRNA splicing, cell adhesion, cell migration, angiogenesis, chemo attraction, immune activities and so on (8, 9). Many researchers have reported overexpression of Gal-3 in different cancers (10, 11). Gal-3 in tumor cells can promote growth and protect the cells from apoptosis induced by chemotherapeutic drugs (11, 12).

Gal-3 overexpression in cancer cells results in its higher secretion and concentration in the circulation of cancer patients (12-19). Increased levels of serum Gal-3 is not limited to cancer as it has been associated with other diseases such as systemic lupus erythematosus, rheumatoid arthritis, Alzheimer's disease and Behçet's disease (15-17). Circulating Gal-3 was positively associated with the type 2 diabetes, which could be reduced by anti-diabetes metformin (19). It was shown, that Gal-3 was associated with fibrosis and inflammation, which has been implicated in development and progression of heart failure (HF) and predicts increased mortality and morbidity in this condition (18). In immune cells, exogenous Gal-3 could induce apoptosis through binding with the CD29/CD7 complex, which was helpful for the immune escape of cancer cells (19).

Extracellular Gal-3 could modulate adhesion of cells to the extracellular matrix, increase tumor cell homotypic aggregation, heterotypic aggregation between tumor cells with blood vascular endothelium and induce angiogenesis *in vivo* and *in vitro* through binding with the corresponding receptors on the cell surface, which are all vital steps in the progression of cancer cell metastasis (1, 8, 9, 20). In addition, after association with the epithelial, macrophages and endothelial cells, Gal-3 could be engulfed into the endosomes (21-23).

Here we would like to clarify the functions and associated mechanisms of circulating Gal-3 on the cell's signal transduction, and report that exogenous Gal-3 selectively activated ERK1/2, but not AKT in a calcium-sensitive and PKC-dependent manner, and the phosphorylation of ERK1/2 was necessary for cell migration. In addition we demonstrated that phosphorylation of paxillin, that was induced by activated ERK1/2 may also be involved in cell migration. These findings were meaningful for probing into exogenous Gal-3 functional mechanisms and finding the potential therapy targets.

## RESULTS

### Exogenous Gal-3 activates MAPK/ERK1/2 but not AKT in a time- and dose-dependent manner

**FIGURE 1:**
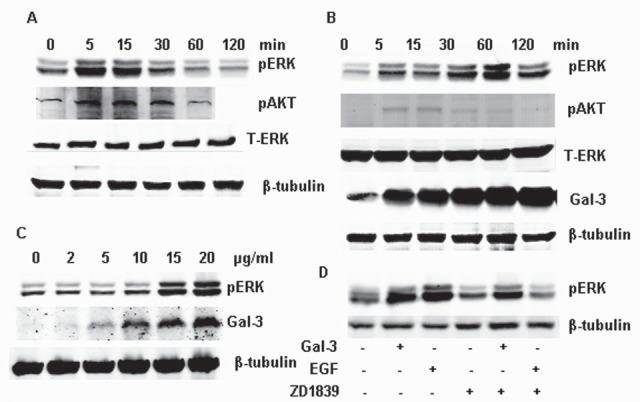
Phosphorylation of ERK1/2 induced by EGF and Gal-3 in HeLa cells HeLa cells were cultured in 6-well cell culture plates, starved by removal of serum for 24 h, and then incubated with EGF (100 ng/ml) and Gal-3 under the stated conditions. After treatment, the cells were collected for western-Blotting using the proper antibodies. A: EGF induced the ERK1/2 phosphorylation. HeLa cells were incubated with EGF (100 ng/ml) for 0, 5, 15, 30, 60, 120 min. B: HeLa cells were incubated with Gal-3 (15 μg/ml) for 0, 5, 15, 30, 60, 120 min. C: HeLa cells were incubated with 0, 2, 5, 10, 15, 20 μg/ml Gal-3 for 30 min. D: HeLa cells were incubated with Gal-3 (15 μg/ml) and EGF (100 ng/ml) in the presence or absence of U0126 and ZD1839 for 30 min, the specific inhibitor of ERK1/2 and EGFR, respectively.

As reported previously, EGF (100 ng/ml) increases phosphorylation of ERK1/2 and AKT in 5 min and returns to a basal level after 1 h, (24), while total ERK level did not change (Figure 1A). Compared to EGF, exogenous Gal-3 induced the phosphorylation of ERK1/2 in a delayed but prolonged way (from 15 min to 120 min); meanwhile, Gal-3 did not induce the phosphorylation of AKT at the corresponding time. The total ERK and AKT also did not change after the treatment with Gal-3 (Figure 1B). The phosphorylation of ERK1/2 induced by EGF and Gal-3 were both aborted by U0126, the specific inhibitor of MEK1/2, suggesting that the signal was transferred through a specific Raf-MEK1/2-ERK1/2 pathway to activate ERK1/2. The phosphorylation of ERK1/2 was concentration-dependent. As shown in figure 1C, phosphorylation increased until the Gal-3 concentration reached 15 μg/ml. Besides, Gefitinib (ZD1839), a novel epidermal growth factor receptor (EGFR) tyrosine kinase inhibitor, could completely inhibit the phosphorylation of ERK1/2 induced by EGF, but could not inhibit the activity induced by Gal-3 (Figure 1D), which further demonstrated that the phosphorylation of ERK1/2 induced by Gal-3 was mediated through different upstream pathways from EGF.

### Phosphorylation of ERK1/2 induced by Gal-3 is CRD dependent and regulated by the N-terminal domain

**FIGURE 2:**
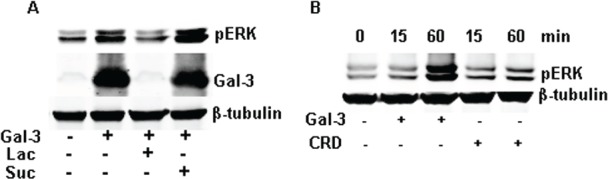
Gal-3 induced the phosphorylation of ERK1/2 dependent on its CRD Starved HeLa cells were incubated with full-length or truncated Gal-3 under the stated condition. The cell lysates were collected for Western-Blotting analysis using the proper antibodies. A: The cells were incubated with Gal-3 (15 μg/ml) in the presence or absence of 50 mM lactose or sucrose. B: The 30 μg/ml CRD domain (including from 111 to 250 animo acids) and full-length Gal-3 (15 μg/ml) were incubated with the cells under the indicated concentration for 15 or 60 minutes.

**FIGURE 3:**
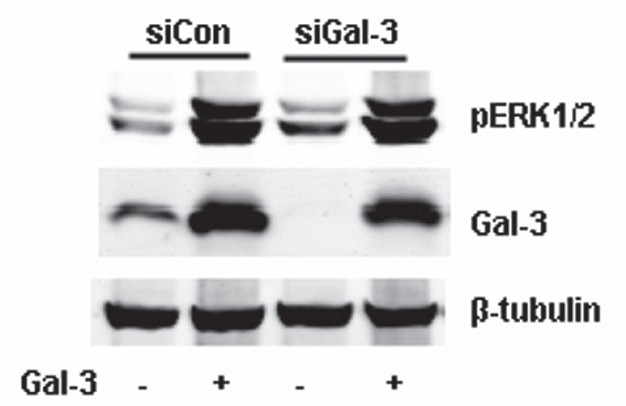
Endogenous Gal-3 knockdown did not affect the phosphorylation of ERK1/2 induced by exogenous Gal-3 HeLa cells were treated with the negative control siRNA (siCon) and Gal-3 siRNA (siGal-3) for 24 h, and then starved for 24h. Gal-3 (15 μg/ml) was added and incubated for 30 min. The cell lysates were collected for Western blotting analysis with the proper antibodies.

Gal-3 is a chimeric gene product composed of a CRD and N-terminal domain, which were implicated in the carbohydrate-recognition and protein-protein interaction (1, 8, 9, 20). Lactose, a potent antagonist of Gal-3, inhibits the carbohydrate-mediated binding of Gal-3 to its ligand(s), (20-23). As shown in figure 2A, lactose inhibits the phosphorylation of ERK1/2 completely, while sucrose (sugar control) did not. To further make clear the potential roles of the CRD and N-terminal domain in the activation of ERK1/2, we have constructed and expressed truncated proteins and checked their ability to phosphorylate ERK1/2. Compared to the full-length Gal-3, the CRD (111 to 250 amino acids) alone resulted in weak phosphorylation of ERK1/2 even at double the concentration and time (Figure 2B). The N-terminal domain (1 to 108 amino acids) failed to induce the phosphorylation of ERK1/2 (data not shown). Thus, intact Gal-3 is required to activate ERK1/2. Next, we knocked down the expression of Gal-3 in HeLa cells and compared with the negative control siRNA (siCon). Gal-3 knockdown diminished the expression of endogenous Gal-3 but did not affect either the basal phosphorylation or the induced phosphorylation of ERK1/2 by exogenous Gal-3 (Figure 3).

### Gal-3 induced ERK1/2 phosphorylation is calcium and Protein Kinase C dependent

**FIGURE 4:**
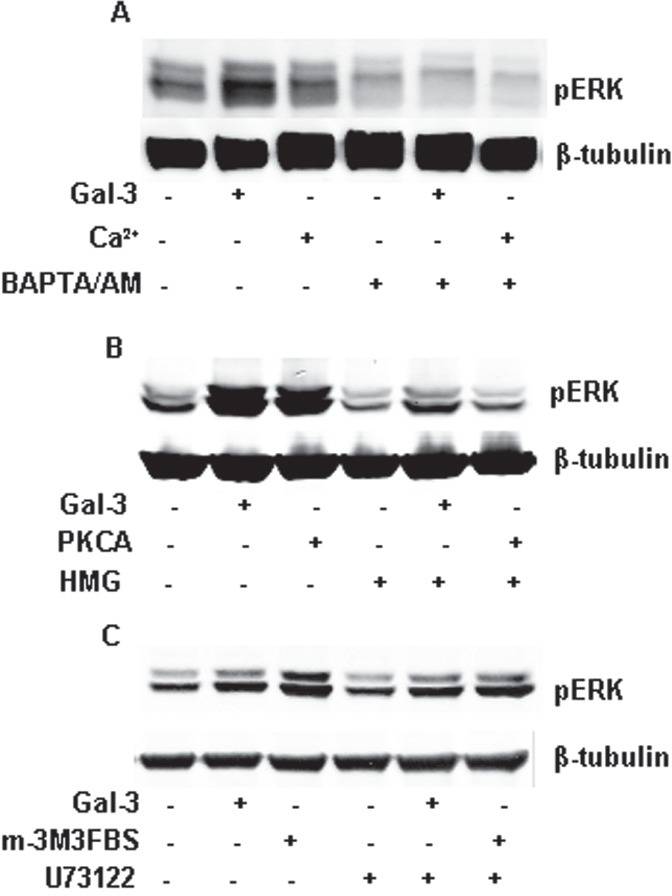
Gal-3 activated PKC, not PLC through intracellular calcium ions Starved HeLa cells were incubated with Gal-3 (15 μ/ml) or some activators in the presence or absence of some inhibitors in serum-less medium for 15 min. The cell lysates were collected and analyzed by Western Blotting using the proper antibodies. A: HeLa cells were incubated with Gal-3 or calcium ions (Ca^2+^) in the presence or absence of BAPTA/AM as indicated. B: HeLa cells were incubated with Gal-3 or PKCA (150 μM, 2-Dioctanoyl-sn-glycerol) in the presence or absence of HMG (150 μM, 1-O-Hexadecyl-2-O-methyl-rac-glycerol) as indicated. C: HeLa cells were incubated with Gal-3 or m-3M3FBS (25 μM) in the presence or absence of U73122 (10 μM) as indicated.

**FIGURE 5:**
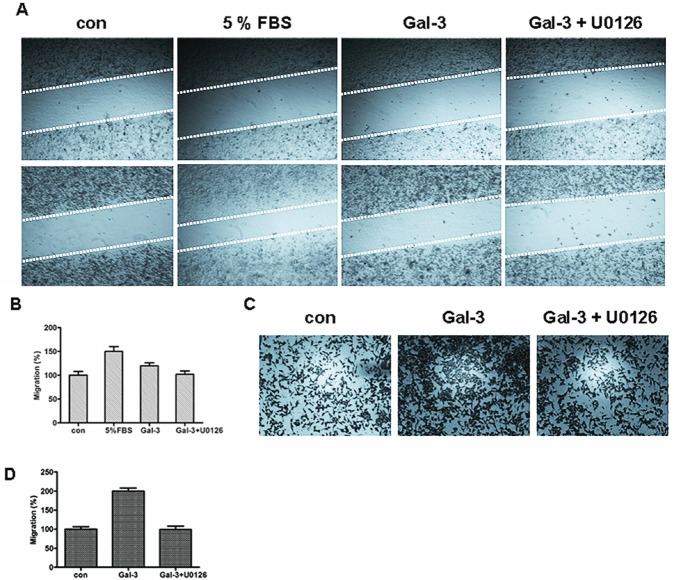
ERK1/2 inhibition decreased Gal-3-induced cell migration A: Starved HeLa cells were incubated with 5% FBS as the positive control, or Gal-3 in the presence or absence of U0126. The upper panels represent the beginning of cell migration, and the lower panels represent the end after 48 h. The relative cell migration ratio was counted (B). C: Starved HeLa cells were seeded in the cell invasion chamber coated with matrigel with Gal-3 in the presence or absence of U0126 for 24 h. The cells were fixed and stained, and then counted under the microscope (D).

Calcium ions are secondary signaling molecules for regulating numerous biological processes (25, 26). Thus, to study their effect in Gal-3 mediated signaling, we demonstrate in figure 4A, that BAPTM/AM, the cell-permeable calcium chelator, could completely inhibit the phosphorylation of ERK1/2 induced by Gal-3. Calcium ions, as an intracellular second messenger, could transfer the signal to the downstream signaling molecules leading to ERK1/2 activation. To further study the downstream signal pathway, we have used specific inhibitors of PLC and PKC kinase, known to be important downstream substrates of calcium ions. As shown in figure 4B, HMG (1-O-Hexadecyl-2-O-methyl-rac-glycerol), the inhibitor of PKC could decrease the phosphorylation of ERK1/2 induced by Gal-3 and PKC activator, (2-Dioctanoyl-sn-glycerol), which meant that PKC was involved in the process of ERK1/2 phosphorylation. Conversely, U73122, the phosphoinositide-specific inhibitor of PLC, did not inhibit the phosphorylation of ERK1/2 induced by Gal-3, but it inhibited the phosphorylation induced by m-3M3FBS, a cell-permeable and specific activator of PLC (Figure 4C), suggesting that circulating Gal-3 may affect the intracellular calcium ions levels leading to activation of PKC. This is supported by the finding showing that activated PKC induces the activation of ERK1/2 through Raf-1 (27), and that PKC activates MEK1/2 directly (28). Cell migration is required for both normal physiological and pathobiological processes (29). Although, Gal-3 was previously reported to regulate cell migration (30-32), by yet to be detrmined patway Here we report, that circulating Gal-3 mediated HeLa cell migration (Figure 5A-5D).

### Gal-3 induced ERK1/2 phosphorylation of by is associated with paxillin phosphorylation

**FIGURE 6:**
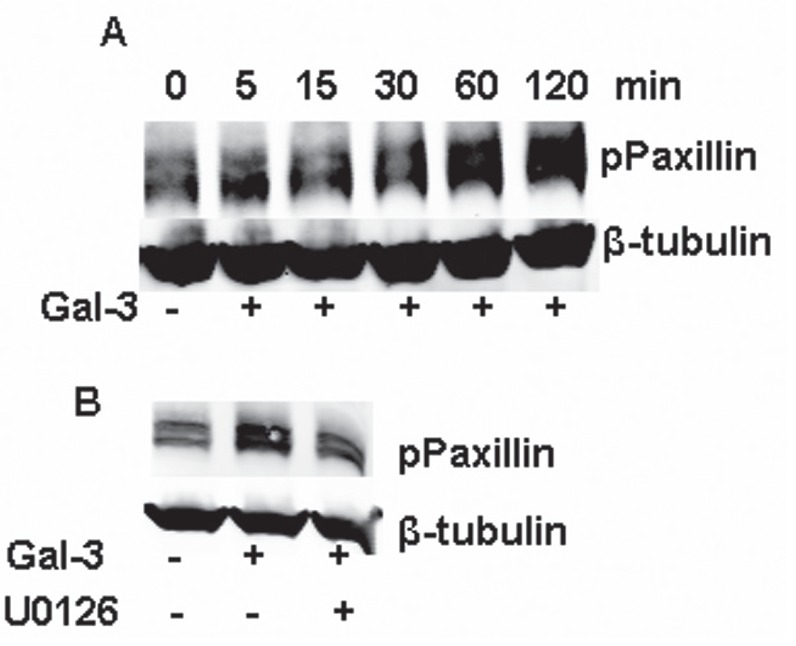
ERK1/2 inhibition aborted the phosphorylation of paxillin induced by Gal-3 A: Starved HeLa cells were incubated with Gal-3 (15 μ/ml) for 0, 5, 15, 30, 60 120 min. B: Starved HeLa cells were incubated with Gal-3 (15 μ/ml) in the presence or absence of U0126 for 60 min. After treatment under the stated conditions, the cell lysates were collected for Western-Blotting analysis.

Paxillin, a focal adhesion and signal transduction adaptor protein, is composed of LD motifs, LIM domains, an SH3 domain-binding site and SH2 domain-binding sites, which mediates the protein-protein interaction involved in focal-adhesion dynamics and cell migration (33). Focal adhesion kinase (FAK) is one of the kinases to phosphorylate paxillin at the sites of tyrosine 31 and tyrosine 118, which could be phosphorylated by ERK1/2. Thus, Gal-3 enhances the phosphorylation of paxillin at the tyrosine 31 in a time-dependent manner (Figure 6A) and the phosphorylation of paxillin is inhibited by U0126 (Figure 6B) suggesting that ERK1/2 inhibition by U0126 results in the inhibition of paxillin phosphorylation.

## DISCUSSION

Gal-3, a chimeric gene-product containing the NWRG anti-death motif of the Bcl-2 family of proteins plays multiple roles in normal development and cancer progression and metastasis. Secreted Gal-3 exhibits multiple autocrine and paracrine properties (1, 8, 9), and serves as biomarker to multiple diseases while its exact function and target(s) remains elusive (13-19). This implies a need for elucidating its function(s) in order to develop a better treatment. Our data identified the pathway induced by exogenous Gal-3 responsible for ERK1/2 phosphorylation, which in turn was required for cell migration. The main signaling mediators in the pathway are summarized: (i) Exogenous Gal-3 (15 μg/ml) activated intracellular calcium ions after incubation for 15 min. (ii) Calcium ions activated PKC, but not PLC, because the phosphorylation of ERK1/2 was inhibited by BAPTA/AM, not U73122. (iii) Activated PKC could directly phosphorylate Raf-1 or MEK1/2, which could explain why Gal-3 just induced the phosphorylation of ERK1/2, but not AKT. The activation of AKT mainly resulted from Ras-PI3K signal pathway; however, in our studies the activation of ERK1/2 resulted from PKC. Therefore, Gal-3 activated ERK1/2 selectively bypassed AKT. Using the cell growth assay we show that Gal-3, unlike EGF, did not increase the rate of proliferation of HeLa cells in serum-less medium, raising the possibility that it is associated with the non-activated AKT, (34) as decrease in cell growth and increase in cell migration protects the cells from apoptosis under starvation (35, 36). (iv) Activated ERK1/2 induced the phosphorylation of paxillin at tyrosine 31, involved in the cell migration, which means that FAK mediates the phosphorylation of paxillin induced by activated ERK1/2. (v) The activity of Gal-3 to phosphorylate ERK1/2 required both the CRD and N-terminal domain, which play very important roles in regulating its carbohydrate-binding with ligands (2), self-association, secretion, and distributions *etc*. Cell migration is one of the critical steps in both plural physiological and pathological processes. Here we show, for the first time, that calcium ions and ERK1/2 are both central signal molecules involved in Gal-3 mediated cell migrations. ERK1/2 catalyzes the phosphorylation of hundreds of cytoplasmic and nuclear substrates including regulatory molecules and transcription factors (37, 38).

In conclusion, circulated Gal-3, is currently used only as a disease biomarker, however, here we have attempted to decipher part of its molecular mechanism and show that Gal-3 specifically activated ERK/1/2, but not AKT through a calcium-sensitive and PKC-dependent signal pathway. The activated ERK1/2 was required for the induction of cell migration directly or indirectly by phosphorylation of paxillin, an adaptor and scaffold protein associated with focal adhesion and cell motility.

## MATERIALS AND METHODS

### Cell lines and regents

HeLa (Human cervix adenocarcinoma epithelial cell line), was cultured in DMEM with high glucose supplemented with 10% fetal bovine serum, 100 μg/mL streptomycin, and 100 units/mL penicillin and was maintained at 37°C in a humidified atmosphere of 95% air and 5% CO_2_. The rat monoclonal antibody against Gal-3 was extracted from the supernatant of hybridoma TIB166 (American Type Culture Collection, Rockville, MD, USA). The monoclonal antibodies against phosphorylated ERK1/2 (E4), total ERK, phosphorylated AKT1/2/3 (Ser473) and the specific siRNA to Gal-3 were purchased from Santa Cruz Biotechnology, Inc. (Santa Cruz, CA.) Rabbit polyclonal antibody against phosphorylated paxillin (pY31) was from BD Biosciences (San Jose, CA). The monoclonal antibody against β-tubulin was obtained from Sigma Chemical (St. Louis, MO USA). Epidermal growth factor (EGF) was purchase from Cell Signaling Technology (Danvers, MA). U0126, the specific inhibitor of MEK1/MEK2, Wortmannin, a specific covalent inhibitor of phosphoinositide 3-kinases, Gefitinib (ZD1839), a selective EGFR inhibitor 1-O-Hexadecyl-2-O-methyl-rac-glycerol, an inhibitor of PKC kinase, and 1,2-Dioctanoyl-sn-glycerol, a cell-permeable PKC activator, were purchased from Cell Signaling Technology. U73122, an inhibitor of phospholipase C, m-3M3FBS, an activator of phospholipase C, and BAPTA/AM, a cell-permeable calcium chelator, were purchased from Calbiochem (Billerica, MA). Lipofectamine RNAiMAX regent was purchased from Invitrogen (Grand Island, NY). Gelcode blue staining regent (24590) and Bio-Rad protein assay kit was both purchased from Bio-Rad Laboratories (Hercules, CA). The lactosyl-sepharose CL 4B was prepared using the lactose and sepharose CL 4B (Sigma) according the protocol published previously (39). Others were of analytical grade or better.

### Preparation of recombinant Gal-3

The recombinant wide-type Gal-3 and truncated Gal-3mutant were constructed in the plasmid pET-22b (+) and expressed in *E.coli* BL21(DE3) through inducement by IPTG. The protein were purified using lactosyl-sepharose CL 4B column according to the protocol reported previously (22). Finally, the protein concentration and purity were analyzed with Gelcode blue staining regent and Bio-Red protein concentration assay kit.

### Analysis of activation of ERK1/2 and AKT by Gal-3

The cells were plated in the 6-well plates and cultured overnight. Then, the medium was exchanged with serum-free medium and cultured for another 24 h. The cells were incubated with or without Gal-3 in the presence or absence of inhibitors. Then, the cells were collected for the SDS-PAGE and Western-blotting using the corresponding antibodies.

### SDS-PAGE and Western-Blotting

The cells, plated in the plates, were collected with the cell scraper in RIPA buffer (20 mmol/L Tris-HCl, pH 7.5, 150 mmol/L NaCl, 1% Triton NP-40, 0.5% sodium deoxycholate, and 0.1% SDS, and adding the Na_3_VO_4_ and PMSF before use) and kept on ice for 30 min. The cell lysate was centrifuged at 14,000 rpm for 15 min at 4°C. The supernatant was removed to a new tube and quantitated. Thirty μg of total protein was loaded and separated in 12% polyacrylamide gel and transferred to PVDF membranes. The membranes were blocked with 0.1% casein in TBS and checked with primary and secondary antibody. Western blotting was performed with the indicated primary antibodies, followed by secondary antibodies conjugated with Alexa 680 (Molecular Probes) or IRDye 800 (Rockland). Fluorescent signals were detected with an Odyssey infrared imaging system. (Licor Lincoln, NE)

### siRNA transfection

siRNA transfection was performed to knockdown Gal-3 according to the manufacturer's instruction for Lipofectamine RNAiMAX regent. After incubation for 24h, the cells were starved by replacing the medium with fresh serum-free medium for another 24h and then the cells were used to perform other treatments.

### Wound-healing Cell migration assay

Cells were seeded into 24-well tissue culture plate in a density, after 24 hours of growth until confluent. The cells were starved with serum-free medium overnight, and they reached approximate 100% confluence as a monolayer. Scratching across the surface of the wells with pipet tips created a cross. The lines were photographed on a microscope under the same configuration at the beginning and at the end, and compared the images to quantify the migration rate of the cells.

### Cell invasion assay

HeLa Cells (5×10^4^) were suspended in 200 μL of serum-free medium after being starved for 24h, and then seeded onto the upper compartment of an 8.0-μm-pore BD Biocoat Matrigel invasion chamber (catalog No. 354480, BD Biosciences, MA, USA). The lower chamber was filled with normal medium containing 10 % FBS. After incubation for 24 h, the medium in the upper chamber was removed and the filters were fixed with 100 % ethanol for15 min and stained with 0.5 % crystal violet for 15 min. The cells remaining on the upper surface of the filter membrane were completely removed by wiping with a cotton swab. The cells that invaded through the membrane were visualized and counted from five randomly selected microscopic fields.

Each experiment was repeated at least three times and representative experiments are presented.
